# Effects of exercise on inflammatory factors and IGF system in breast cancer survivors: a meta-analysis

**DOI:** 10.1186/s12905-022-02058-5

**Published:** 2022-12-08

**Authors:** Yanan Zhou, Ningxin Jia, Meng Ding, Kai Yuan

**Affiliations:** 1 College of Physical Education, Shandong Normal University, Jinan, 250014 China; 2 Department of General Surgery, The First Affiliated Hospital of Shandong First Medical University, Jinan, 262799 Shandong China

**Keywords:** Breast cancer, Exercise, Inflammatory factors, IGF system

## Abstract

**Background:**

At present, there are multiple hypotheses regarding the mechanisms underlying the effect of exercise on the postoperative inflammatory factors and the IGF system among breast cancer patients, especially. To determine the underlying mechanisms, prevent the recurrence of breast cancer and improve its prognosis, this paper will systematically evaluate the impact of exercise on inflammatory factors and the IGF system in breast cancer survivors.

**Methods:**

The PubMed, Embase, Web of Science, CNKI, Wanfang and VIP (Chinese scientific and technical journals) databases were systematically searched until April 2021. The search terms included 'exercise', 'inflammatory factor', 'IGF system' and 'breast cancer'. A total of 1066 relevant articles were retrieved. The articles were screened according to inclusion and exclusion criteria, such as study population, intervention method and type of experiment, and 11 articles were ultimately included. All statistical results were analysed using STATA 14.0 and Rstudio 4.1.1.

**Results:**

We found that exercise significantly reduced the level of IGF-1 (WMD, -19.947 ng/ml; 95% CI, -22.669 to -17.225; *P* = 0.000). Subgroup analysis showed that in the studies with an intervention period > 12 weeks, exercise significantly reduced IL-6 levels (WMD, -0.761 pg/ml; 95% CI, -1.369 to -0.153; *p* = 0.014), while in the studies with an intervention period ≤ 12 weeks, exercise significantly reduced CRP (WMD, -2.381 mg/L; 95% CI, -4.835 to 0.073, *P* = 0.001) and IL-10 levels (WMD, -7.141 pg/ml, 95% CI, -10.853 to -3.428; *P* = 0.000). In addition, aerobic exercise plus resistance training significantly reduced IL-6 levels (WMD, -1.474 pg/ml; 95% CI, -1.653 to -1.296; *P* = 0.000). The results of the sensitivity analysis showed that after excluding the studies with high heterogeneity, exercise significantly reduced the TNF-α levels in patients with breast cancer (WMD, -1.399 pg/ml; 95% CI, -1.718 to -1.080; *P* = 0.000).

**Conclusion:**

Exercise reduces the postoperative levels of IGF-1, IL-6, CRP, IL-10 and TNF-α among patients with breast cancer, which may have a significant impact on inhibiting breast cancer recurrence and improving its prognosis. Future studies should examine the effects of different durations and types of exercise to develop individualized exercise prescriptions for breast cancer patients.

**Supplementary Information:**

The online version contains supplementary material available at 10.1186/s12905-022-02058-5.

## Background

Globally, breast cancer is the most common cancer as well as the most common cause of cancer-related death in women [1]. Therefore, how to reduce the recurrence rate of breast cancer and improve the survival rate of breast cancer patients is the main research focus. At present, several mechanisms have been proposed regarding the aetiology and progression of breast cancer [2], including inflammatory factors and the IGF system.

Inflammatory factors are synthesized by fibroblasts and endothelial cells, and they are dependent on the tumour microenvironment to regulate the survival, proliferation, differentiation, activation, migration and death of tumour cells [3]. The impact of chronic inflammation on tumorigenesis and the tumour microenvironment is widely recognized as playing a key role in the risk of cancer development, progression and recurrence [4]. The main inflammatory factors associated with tumours include interleukin 6 (IL-6), interleukin 10 (IL-10), interleukin 1β (IL-1β), tumour necrosis factor alpha (TNF-α) and C-reactive protein (CRP). Studies have shown that systemic inflammation – characterized by elevated levels of TNF-α, IL-6 and CRP – is associated with an increased risk of breast cancer progression and death [5–8]. In addition, elevated serum concentrations of IL-6, IL-10, IL-1β, and TNF-α are found in a variety of cancer types, including breast cancer [3, 9–11].

The insulin-like growth factor (IGF) system is made up of ligands, binding proteins and receptors that regulate important physiological and pathological processes. The ligands include the three major ligands, IGF-1, IGF-2 and insulin; the IGF receptors include the insulin receptor (IR), IGF-1R, IGF-2R and insulin-related receptor (IRR), and at least six circulating IGF binding proteins (IGFBPs) [12]. The biological activity of the IGF system depends on the binding of ligands to their cognate receptors; the cognate receptors of IGF-1, IGF-2 and insulin are IR, IGF-1R and IGF-2R, respectively. IGF-1R is expressed in a variety of human tissues, and its activation regulates cell survival, proliferation, differentiation and protein synthesis [13]. IGFs effectively bind to IGF-1R and activate pathways associated with cell proliferation, and the interaction between IGFs and IGF-1R promotes tumour cell growth and metastasis [14, 15]. The expression of insulin-like growth factor-1 (IGF-1) is higher in the sera of breast cancer patients than in those of benign tumour patients. Therefore, it has been suggested that elevated IGF-1 levels may predict tumour progression and metastasis [16]. IGFBPs can regulate IGF levels in serum and tissues, thereby promoting apoptosis of cancer cells. For example, IGFBP-3 is a highly relevant IGF-1 binding protein. IGFBP-3 can inhibit IGF-1 binding to IGF-1R (IGF-1 receptor) through competitive binding to IGF-1 [17, 18]. Studies have shown that elevated circulating levels of IGF-1 and reduced levels of IGFBP-3 are associated with an increased risk of premenopausal breast cancer [19–21].

Given the impact of inflammatory factors and the IGF system on breast cancer, improving the inflammatory status and IGF system in breast cancer survivors may be a new way to reduce the risk of breast cancer recurrence. Studies have shown that exercise can improve the tumour microenvironment in breast cancer patients. Physical activity is associated with lower levels of various proinflammatory cytokines [22, 23]. Epidemiological studies have shown that a highly physically active lifestyle is associated with lower circulating levels of TNF-α, IL-6 and CRP, independent of age, sex, body mass index or blood glucose [24]. High levels of physical activity are associated with 20%-60% lower levels of peripheral inflammatory mediators than a sedentary lifestyle [25]. In addition, exercise was also associated with levels of IGF-I and IGFBPs. [26, 27]. Physical activity reduces IGF-1 levels and increases IGFBP-3 levels [28], which in turn inhibits the IGF signalling pathway. In addition, there is an association between exercise and breast cancer-specific mortality and all-cause mortality. The risk of specific mortality and all-cause mortality is lower in breast cancer patients who participate in physical activity [29] [30–32]. There is growing evidence that exercise improves levels of inflammatory factors and the IGF system in breast cancer patients, reduces the risk of breast cancer recurrence and improves patient survival. However, the results among individual studies are inconsistent. Sprod et al. [33] found no significant changes in insulin-like growth factor binding protein-1 (IGFBP-1) and IGFPB-3 in 21 breast cancer survivors after a 12-week tai chi intervention. A meta-analysis also came to a different conclusion, with a significant reduction in IGFBP-3 levels following the exercise intervention [34]. In addition, another meta-analysis found that exercise interventions increased IL-6 and IL-10 levels [35]. However, these studies differed in terms of the time of the intervention and the mode of intervention, which may have contributed to the differences in the findings. The American College of Sports Medicine (ACSM) suggests that breast cancer patients should participate in at least 150 min of moderate or 75 min of vigorous aerobic exercise per week and strength training at least two days per week [36]. However, detailed information on exercise is still lacking, and further research is needed on which types or durations of interventions are most beneficial for breast cancer patients. Therefore, this meta-analysis will focus on the effects of exercise on IL-6, IL-10, IL-1β, CRP, TNF-α, IGF-1 and IGFBP-3 levels in breast cancer patients. The effects of different intervention durations and modalities on inflammatory factors and the IGF system will be further explored using subgroup analysis. Additionally, this meta-analysis aims to provide evidence regarding the effect of exercise on preventing the recurrence of breast cancer and improving its prognosis. Moreover, this study aims to provide a theoretical basis for the development of exercise interventions for breast cancer patients.

## Methods

### Search strategy

This meta-analysis was conducted in accordance with the PRISMA guidelines for evidence-based medicine. The PubMed, Embase, Web of Science, CNKI, Wanfang and VIP (China Science and Technology Journal) databases were systematically searched until April 2021. The search terms included "physical activity or exercise or sport or training" and "breast cancer or breast tumour or breast oncology" and "inflammatory or IL-6 or IL-10 or IL-1β or CRP or TNF-α or IGF or IGF-1 or IGFBP-3" (refer to supplemental 1). The protocol for this systematic review was registered on INPLASY (ID = INPLASY2021100101) and is available in full on the inplasy.com (https://doi.org/10.37766/inplasy00000000).

### Eligibility criteria

All the retrieved literature was screened based on the inclusion and exclusion criteria. The screening criteria were as follows:

Inclusion criteria: (1) subjects: breast cancer survivors; (2) intervention measures: exercise intervention (including aerobic exercise, resistance exercise, resistance exercise combined with aerobic exercise, high-intensity interval training, etc.); (3) study content: effects of exercise group (physical activity) and control group on inflammatory factors or IGF system in patients with breast cancer; (4) intervention time: postoperative and 1 month to 5 years posttreatment; (5) study type: randomized controlled trial and include at least one blank group.

Exclusion criteria: (1) the intervention measures were not simple exercise but instead entailed exercise intervention combined with other therapies (such as exercise combined with diet or exercise combined with drugs); (2) only experimental design, there was no specific intervention process; (3) review study, duplicate publication and or unable to obtain the full text; and (4) the main research indicators were not consistent or the data were incomplete.

### Data extraction

Two evaluators independently carried out retrieval and screening and then checked and compared the results. If there were any differences, they were decided by a third party. The extracted data included basic information (title, original study author, year of publication, country), basic characteristics of the subjects (sample size, mean age, sex, cancer stage, etc.), characteristics of the exercise intervention (exercise type, exercise frequency, exercise time, intensity, etc.), characteristics of the control group (routine treatment, placebo control, etc.) and the outcome indicators (the changes in indicators before and after intervention, *P* value and/or CI).

### Risk of bias assessment

The Cochrane manual evaluation standard (version 5.0.2) was used to comprehensively evaluate the literature quality and objectively evaluate whether there were methodological errors and subjective biases. In summary, the risk of bias was assessed in the following six domains: (1) random sequence generation; (2) allocation concealment; (3) blinding; (4) incomplete outcome data; (5) selective outcome reporting; and (6) other sources of bias. The above criteria were used to evaluate the quality of the article, and the results were judged as "low risk", "high risk" or "unclear risk".

### Outcome indicators

The outcome indicators included the levels of IL-6, IL-10, IL-1β, CRP, TNF-α, IGF-1 and IGFBP-3 in each group.

### Statistical analysis

Stata 14.0 and R Studio 4.1.1 software [37] were used to perform a meta-analysis of the differences between endpoint and baseline indicators (formula: SD_change_ = √SD_1_^2^ + SD_2_^2^-(2*R*SD_1_*SD_2_), where *R* = 0.5). The extracted data were all continuous variables, and the individual test units were converted. Since the study of Karimi et al. [38] only mentioned endpoint indicators, we combined their postintervention data with differential data from other studies, in accordance with previous research [39]. The mean ± SD was chosen as the standard scale of effect in the article, and the statistics were expressed as weighted mean differences (WMDs) with 95% confidence intervals (CIs). *P* < 0.05 was considered to indicate a statistically significant difference. The I^2^ value was used to assess heterogeneity analysis among studies. When I^2^ = 0, no heterogeneity was observed among studies, and a fixed effects model was used; when I^2^ ≥ 50%, heterogeneity among studies was observed, and a random effects model was used. When there was heterogeneity between studies, subgroup analysis was used to analyse the sources of heterogeneity, such as grouping different intervention periods and types for the computational analysis of I^2^ values. In addition, to increase the credibility of the meta-analysis, sensitivity analysis was conducted to analyse whether there was a significant effect of any individual article on the pooled results.

## Result

### Article search results

**Fig. 1:**
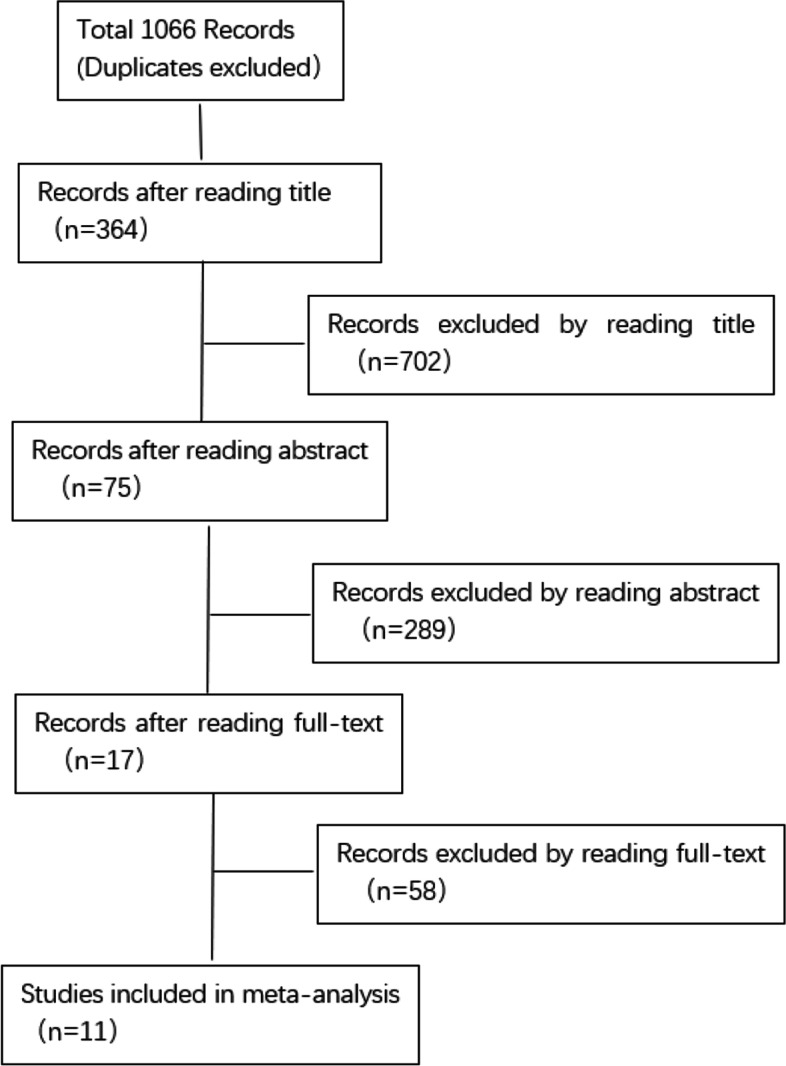
Studies selection flow diagram

After searching by subject terms and excluding duplicates, 1066 relevant articles were retrieved. After reading the titles, 364 articles remained. In addition, 289 articles were manually excluded after screening the abstracts, leaving 75 articles. A total of 17 articles met the criteria after reading the full text, of which 6 articles did not mention specific data. Therefore, 11 articles were ultimately included. A flow diagram for study selection is presented in Fig. 1.

### Characteristics of the selected studies

Table 1 summarizes the details of the 11 included articles. In brief, 11 articles were reported from 10 RCTs. Of the 10 trials, 8 studies were 2-arm randomized controlled trials [40] with exercise groups and control groups; one study was a 3-arm randomized controlled trial including two exercise groups (high-intensity interval exercise plus resistance exercise, high-intensity interval exercise plus aerobic exercise) and control groups, which were analysed separately in the text; another study was a 4-arm randomized controlled trial including an exercise group, a ginger-taking group, the taking ginger plus exercise group and the placebo group, and then we extracted data only from the exercise and placebo groups. Among the 11 articles, nine studies examined IL-6 [33, 40–47], five studies examined CRP [38, 42, 44–46], five studies examined TNF-α [41–43, 45, 47], four studies examined IL-10 [38, 40, 41, 43], three studies examined IGF-1 [33, 45, 48] and IGFBP-3 [33, 45, 48], and 1 study examined IL-1β [41] (not analysed). The number of individuals in each study ranged from 16 to 240, with a total number of 696 individuals. The intervention types included aerobic, resistance training, aerobic plus resistance training, yoga, water-based exercise, HIIT plus aerobic exercise, HIIT plus resistance training, and tai chi. The intervention settings included supervised, home-based or mixed. The exercise intensity included moderate, vigorous, or increasing. The exercise frequency ranged from 1 session per week to 5 sessions per week. The intervention period ranged from 8 to 24 weeks, and the exercise duration ranged from 30–90 min.

**Table 1:** Characteristics of the selected studies

Author(year)	Deign	Participants	Intervention	Adherence	biomarkers	Other variables
Irwin, etc.* (2009) [48]	Two-arm RCT. AT;UC	*N* = 75, stage I-IIIA BCS, AT mean age: 56.4 ± 9.5	AT, 24wks, 5 d/wk, 150 min/wk		IGF-1,IGF-3	Insulin, etc
Lisa, etc. (2011) [33]	Two-arm RCT.TCC; SST	*N* = 21, I-IIIB BCS, TCC mean age: 54.33 ± 10.64	TCC, 12 wks, 3 d/wk,60 min	72%	IL-6 IGF-1 IGFBP-3	HRQOL, etc
Gomez, etc.(2011) [41]	Two-arm RCT.AT + RT;UC	*N* = 16, stage I-II BCS, mean age:50 ± 5	AT + RT 8 wks, 3 d/wk, 90 min, supervised	91%	IL-1, IL-10 IL-1β IL-6, IL-10/TNF-α TNF-α,	VO2max weight, etc
Rogers, etc. (2012) [43]	Two-arm RCT.AT + RT;UC	*N* = 28, I-IIIA BCS, mean age: 56 ± 10.5	AT + RT, 12 wks, 2 d/wk, 150 min/wk supervised		IL-10, IL-6, Il-1β, IL-6/IL-10,TNF-α/IL-10	BMI, Fatigue, etc
Jones, etc.* (2012) [42]	Two-arm RCT AT;UC	*N* = 75, stage I-IIIA BCS, AT mean age: 56.4 ± 9.5	AT, 24 wks, 5 d/wk, 150 min/wk		CRP、IL-6、TNF-α	BMI, etc
Bower, etc. (2013) [44]	Two-arm RCT. yoga; HE	*N* = 31, stage I-II BCS; mean age: 54 ± 5.7	Yoga,12w ks, 2 d/wk, 90 min, supervised	78%	IL-6, CRP	Fatigue, depression, etc
Karimi, etc. (2015) [38]	Four-arm RCT. exercise; placebo; GS; exercise + GS	*N* = 40, stage I-II BCS, exercise mean age: 47.3 ± 8.1	Water-Based Exercise, 6 wks, 4 d/wk, 40–80 min		IL-10, hs-CRP	Insulin, etc
Christina, etc. (2017) [45]	Two-arm RCT.AT + RT; delayed intervention control	*N* = 20, stage I-III BCS, AT + RT mean age: 53.0 ± 10	AT + RT, 16 wks, 3 d/wk 80 min, supervised	97%	CRP, IL-6	Body composition, etc
Christina, etc. (2018) [46]	Two-arm RCT. AT + RT; UC	*N* = 100, stage BCS, mean age: 53.5 ± 10.4	AT + RT, 16 wks, 3 d/wk AT:150 min/wk, supervised	95%	IGF-1, CRP, IL-6, TNF-α	Weight, BMI, etc
Kim, etc. (2019) [47]	Two-arm RCT. exercise; no exercise	*N* = 50, stage I-IIIA BCS, exercise mean age: 49.95 ± 8.12	stretching and resistance exercise, 12 wks, 1d/wk	90%	IL-6,TNF-α	Fatigue, etc
Anouk E, etc. (2020) [40]	Three-arm RCT.HIIT + RT;HIIT + AT;UC	*N* = 240, stage I-IIIA BCS, mean age: RT: 52.2 ± 10.1 AT: 53.9 ± 7.4	HIIT + RT; HIIT + AT, 16 wks, 2 d/wk,60 min	RT:79.5%; AT:82.1%	IL-6, TNF-α	Muscular strength, etc

*Abbreviations*: *AT* Aerobic training, *RT* Resistance training, *HE* Health education, *GS* ginger supplement, *SST* standard support therapy, *UC* Usual care, *TCC* Tai chi chuan

^*^two articles from the same experiment

### Methodological quality of selected studies

**Fig. 2:**
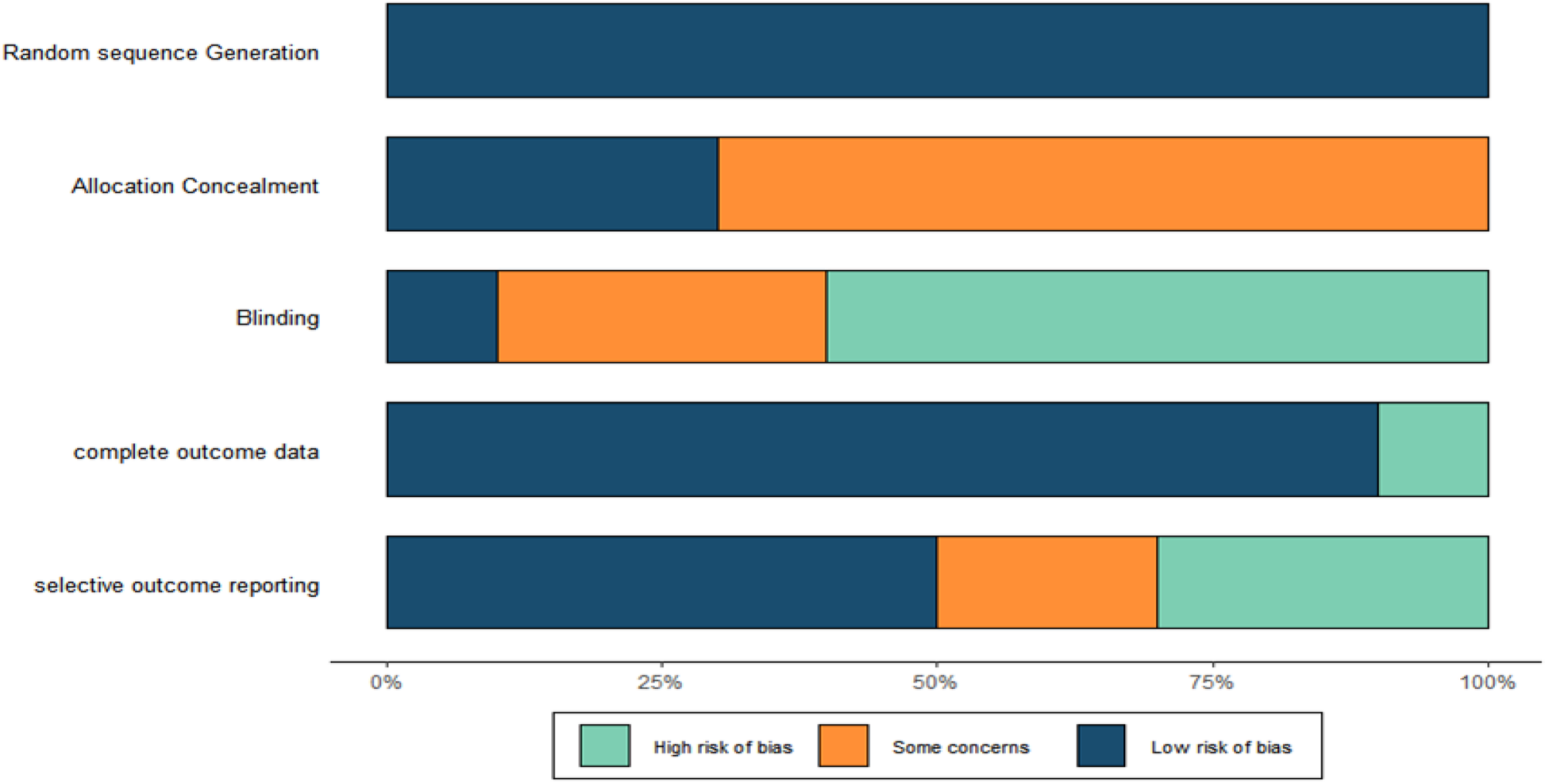
Risk of bias

**Fig. 3:**
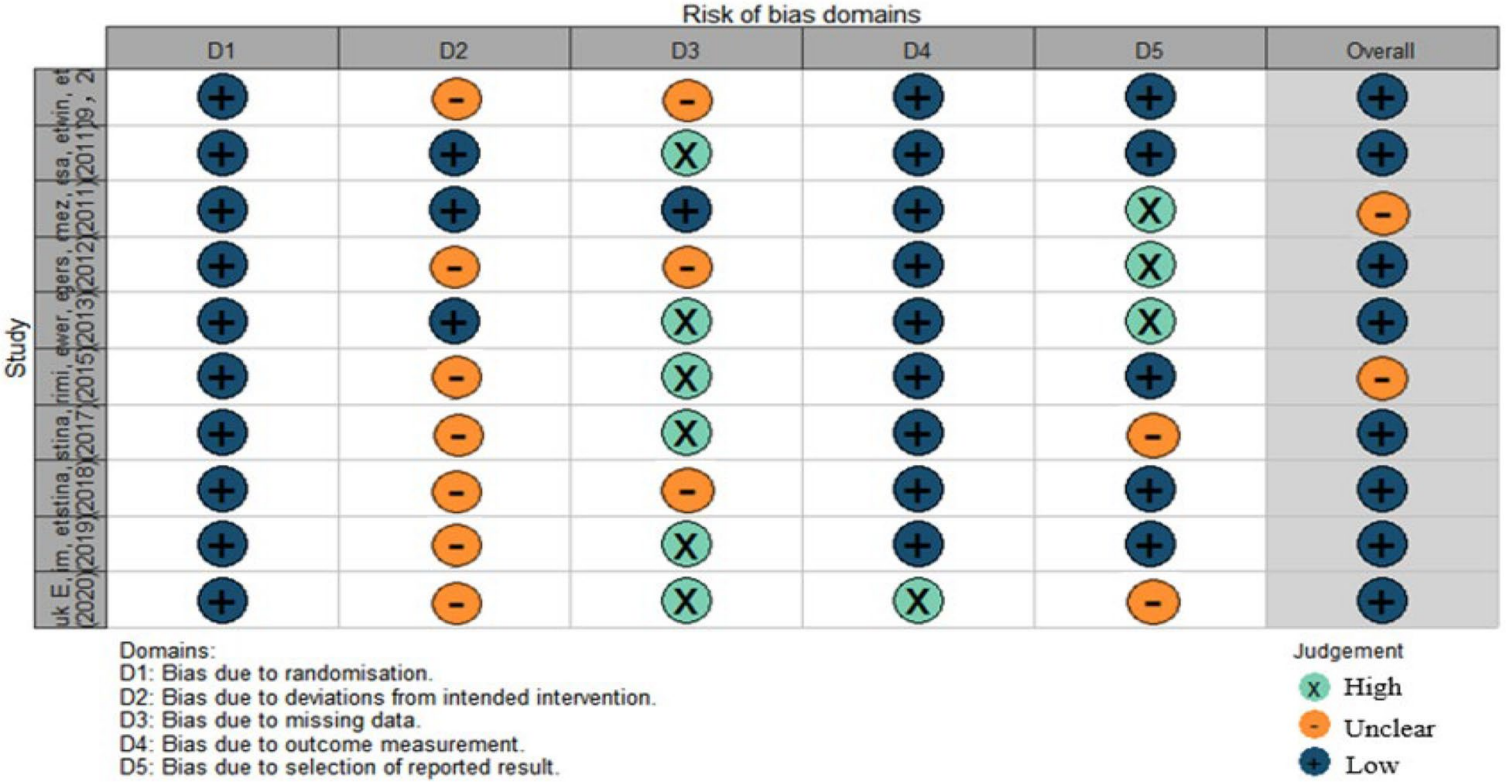
Risk of bias domains. Study (Top down): Irwin, etc.(2009,2012); Lisa, etc.(2011); Gomez, etc.(2011); Rogers, etc.(2012);Bower, etc.(2013); Karimi, etc. [38]; Christina, etc. (2017); Christina, etc. (2018); Kim, etc. (2019); Anouk E, etc. (2020)

All 10 randomized controlled trials mentioned randomization. Only 2 studies mentioned the method of randomization. Three studies mentioned allocation concealment. Only one study implemented blinding; because the interventions were exercise, blinding was difficult to implement. Seven studies mentioned participant dropout or loss to follow-up. The methodological quality of the studies is shown in Figs. 2 and 3.

### Results of meta-analyses

#### The effect of exercise on IL-6 levels

**Fig. 4:**
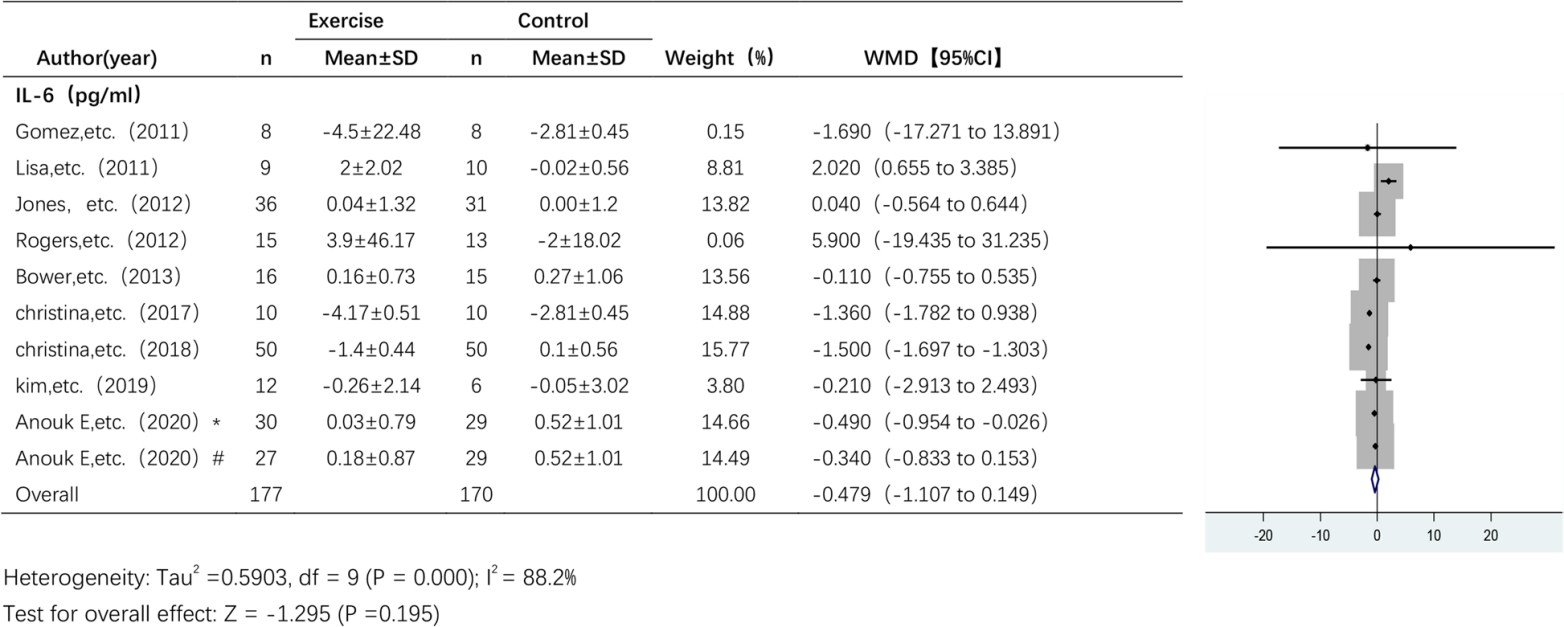
Forest plot of comparison for IL-6 levels. Anouk E, etc. (2020)* RT + HIIT group; Anouk E, etc. (2020)#AT + HIIT group

Nine studies analysed the effect of exercise on IL-6 levels across a total of 318 participants (Fig. 4). A random effects model was used due to the high heterogeneity among the pooled studies (I^2^ = 88.2%). The analysis showed a trend towards a decrease in IL-6 levels following the exercise intervention, but there was no statistically significant difference in IL-6 levels between the exercise group and the control group (WMD, -0.479 pg/ml; 95% CI, -1.107 to 0.149, *p* = 0.195).

#### The effect of exercise on CRP levels

**Fig. 5:**
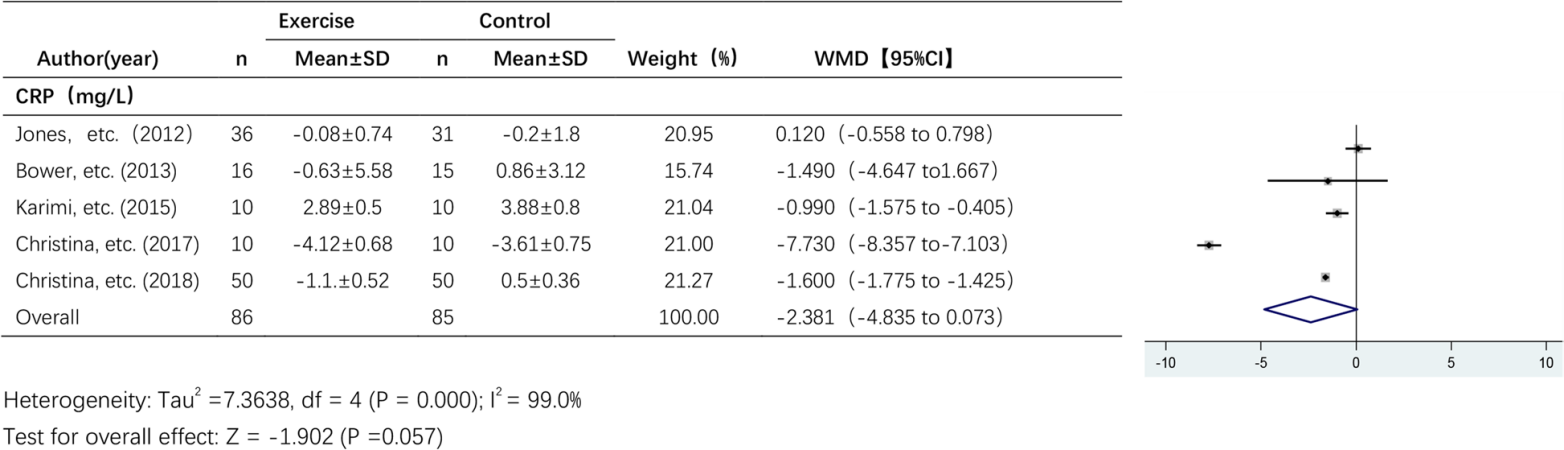
Forest plot of comparison for CRP levels. Karimi, etc. [38]: endpoint indicators

Five studies reported the effects of exercise on CRP levels (Fig. 5). A random effects model was used due to the high heterogeneity among the pooled studies (I^2^ = 99.0%). The results showed a trend towards a decrease in CRP levels after the exercise intervention, but the effect of exercise on CRP levels was not statistically significant (WMD, -2.381 mg/L; 95% CI, -4.835 to 0.073; *P* = 0.057).

#### The effect of exercise on TNF-α levels

**Fig. 6:**
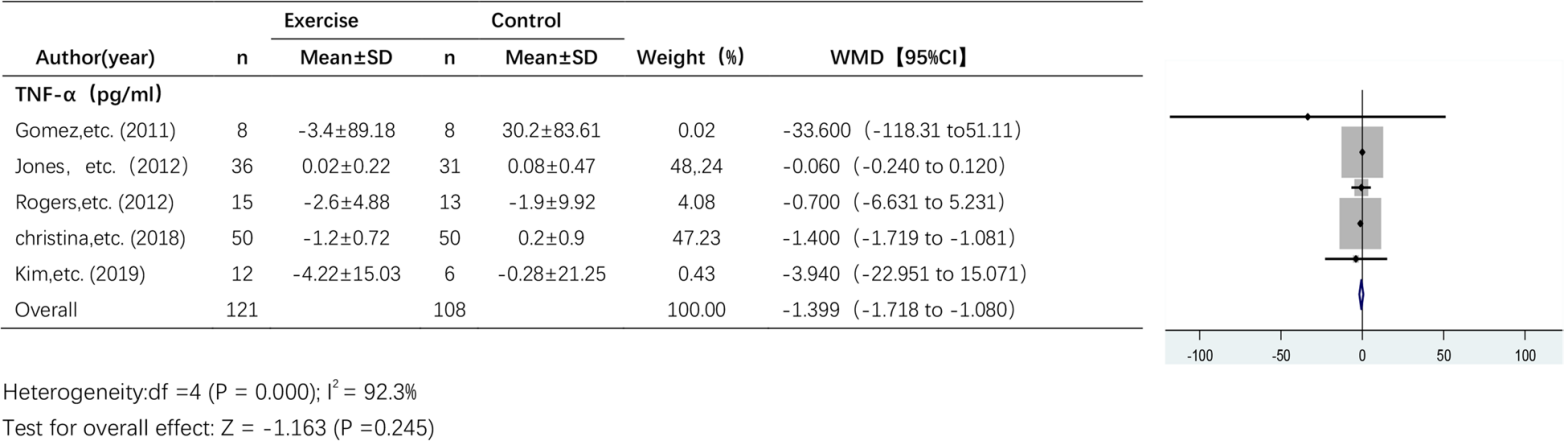
Forest plot of comparison for TNF-α levels

Five studies reported the effect of exercise on TNF-α levels (Fig. 6). A random effects model was used due to the high heterogeneity among the pooled studies (I^2^ = 92.3%). The results showed a trend towards a decrease in TNF-α levels after exercise intervention, but the effect of exercise on TNF-α levels was not statistically significant (WMD, -1.399 pg/ml; 95% CI, -1.718 to -1.080; *P* = 0.245).

#### The effect of exercise on IL-10 levels

**Fig. 7:**
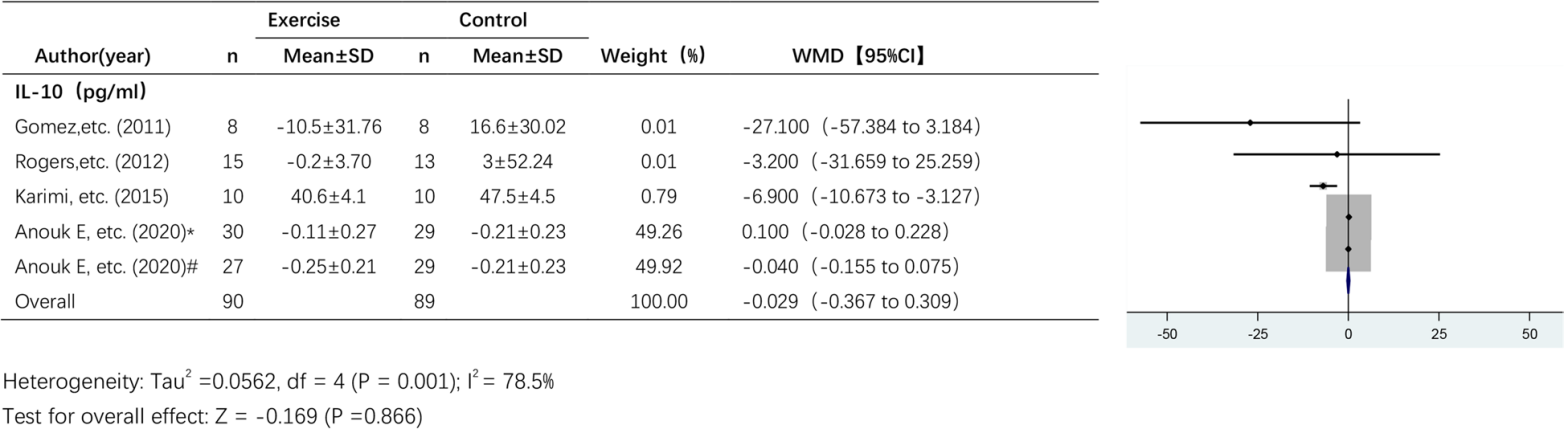
Forest plot of comparison for IL-10 levels. Abbreviations: Karimi et al. [38] are endpoint indicators; Anouk E, etc. (2020)* RT + HIIT group; Anouk E, etc. (2020)#AT + HIIT group

Four studies analysed the effect of exercise on IL-10 levels (Fig. 7) across a total of 150 participants. A random effects model was used due to the high heterogeneity among the pooled studies (I^2^ = 78.5%). The results showed a trend towards a decrease in IL-10 levels after exercise intervention, but the effect of exercise on IL-10 levels was not statistically significant (WMD, -0.029 pg/ml; 95% CI, -0.367 to 0.309; *P* = 0.866).

#### The effect of exercise on IGF-1 levels

**Fig. 8:**
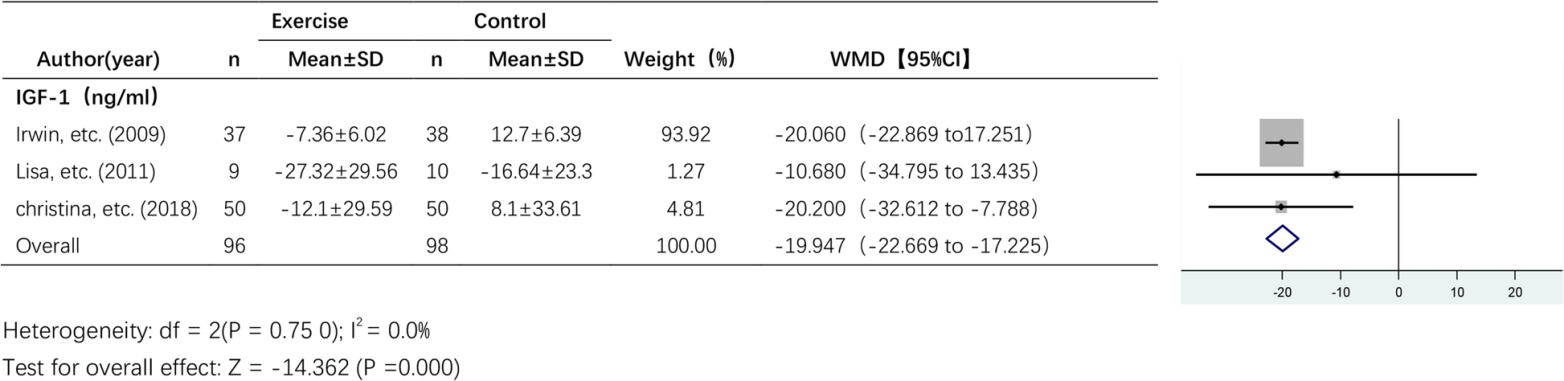
Forest plot of comparison for IGF-1 levels

Three studies reported the effect of exercise on IGF-1 levels (Fig. 8). A fixed effects model was used due to the lack of statistical heterogeneity among the pooled studies (I^2^ = 0.0%). I^2^ was 0.0%, and there was no statistical heterogeneity, so a fixed effect model was used. The results showed a significant effect of exercise on IGF-1 levels (WMD, -19.947 ng/ml; 95% CI, -22.669 to -17.225; *P* = 0.000).

#### The effect of exercise on IGFBP-3 levels

**Fig. 9:**
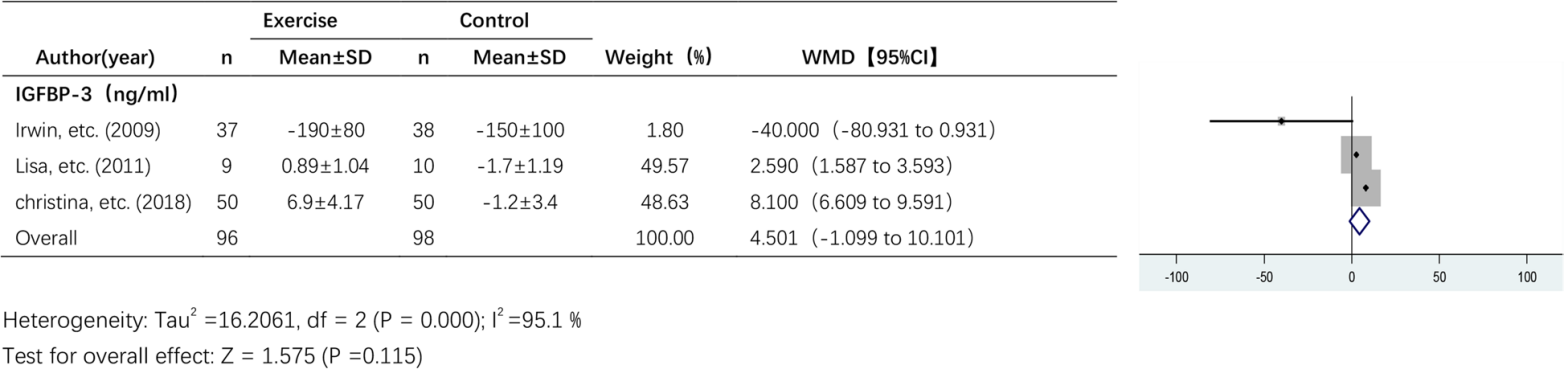
Forest plot of comparison for IGFBP-3 levels

Three studies reported the effect of exercise on IGFBP-3 levels (Fig. 9). A random effects model was used due to the high heterogeneity among the pooled studies (I^2^ = 95.1%). I^2^ was 95.1%, and there was a high degree of heterogeneity between studies, so a random effects model was used. There was an increasing trend in IGFBP-3 levels after the exercise intervention, but the effect of exercise on IGFBP-3 levels was not significant (WMD, 4.501 ng/ml; 95% CI, -1.099 to 10.101; *P* = 0.115).

### Subgroup analysis

Considering the large differences in the intervention period among all studies, we grouped them according to the intervention periods: > 12 weeks and ≤ 12 weeks. To analyse the heterogeneity of the included studies, we performed subgroup analyses for some of the studies, as shown in Table 2. (1) Subgroup analysis of the effect of exercise on IL-6 levels: A total of 9 studies examining IL-6 levels were included, including 5 studies with an intervention period ≤ 12 weeks and 4 studies with an intervention period > 12 weeks. The results of the subgroup analysis showed that the effect of exercise on IL-6 levels was significant in studies with an exercise intervention period > 12 weeks (WMD, -0.761 pg/ml; 95% CI, -1.369 to -0.153; *p* = 0.014), while in studies with an exercise intervention period ≤ 12 weeks, there was no significant effect of exercise on IL-6 levels (WMD, 0.615 pg/ml; 95% CI, -0.763 to 1.993; *p* = 0.382). (2) Subgroup analysis of the effect of exercise on CRP and IL-10 levels: In studies with an intervention period ≤ 12 weeks, exercise had significant effects on CRP (WMD, -2.381 mg/L; 95% CI, -4.835 to 0.073, *p* = 0.001) and IL-10 levels (WMD, -7.141 pg/ml; 95% CI, -10.853 to -3.428; *P* = 0.000).

**Table 2:** Subgroup analysis of different intervention periods

group	standard	total	WMD	95% CI	P	I^2^	P (heterogeneity)
IL-6	≤ 12wks	5	0.615	-0.763 to 1.993	0.382	50.1%	0.091
	> 12wks	4	-0.761	-1.369 to-0.153	0.014	91.3%	0.000
CRP	≤ 12wks	2	-2.381	-4.835 to 0.073	0.001	0.0%	0.760
	> 12wks	3	-3.068	-6.908 to 0.772	0.117	99.5%	0.000
TNF-α	≤ 12wks	3	-1.132	-6.782 to 4.517	0.694	0.0%	0.716
	> 12wks	2	-0.723	-2.036 to 0.590	0.280	98.0%	0.000
IL-10	≤ 12wks	3	-7.141	-10.853 to-3.428	0.000	0.0%	0.415
	> 12wks	1*#	0.027	-0.110 to 0.164	0.698	60.7%	0.111

^*^Anouk E, etc. (2020) RT + HIIT group; #Anouk E, etc. (2020) AT + HIIT group

In addition, we performed a subgroup analysis based on intervention type, but due to the number of articles for each outcome, we only examined studies that reported IL-6 levels. The results of the subgroup analysis are shown in Table 3. Among the studies that reported IL-6 levels, there were 4 studies that used aerobic plus resistance training, two studies that used Tai Chi and yoga, one study that used aerobic exercise, one study that used resistance exercise, one study that used high-intensity interval exercise plus resistance exercise, and one study that used high-intensity interval exercise plus aerobic exercise. Subgroup analyses showed that the effect of aerobic plus resistance exercise on IL-6 levels was significant (WMD, -1.474; 95% CI, -1.653 to -1.296; *P* = 0.000).

**Table 3:** Subgroup analysis of the effect of different intervention types on IL-6 levels

group	total	WMD	95% CI	P	I^2^	P (heterogeneity)
AT + RT	4	-1.474	-1.653 to -1.296	0.000	0.0%	0.879
Mindbody	2	0.867	-1.214 to 2.947	0.414	86.9%	0.006
AT	1	0.04	-0.564 to 0.644	0.897		
RT	1	-0.210	-2.913 to 2.493	0.879		
HIIT + RT	1	-0.490	-0.954 to -0.026	0.038		
HIIT + AT	1	-0.240	-0.833 to 0.153	0.176		

*Abbreviations:* *AT + RT* Aerobic training + Resistance training; Mindbody: Taiji, yoga

### Sensitivity analysis

**Fig. 10:**
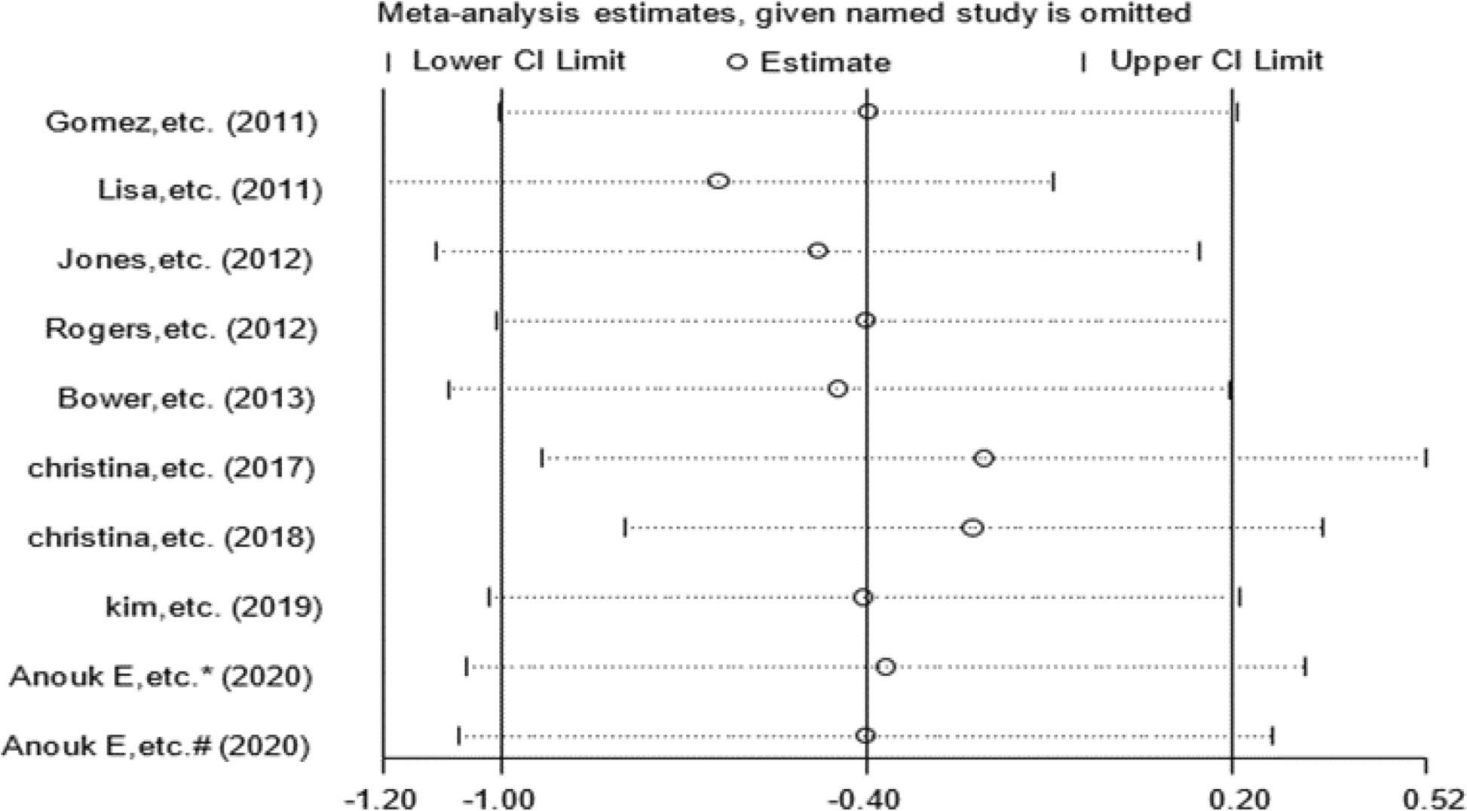
Sensitivity analysis of IL-6 levels. Anouk E, etc. (2020)* RT + HIIT group; Anouk E, etc. (2020)# AT + HIIT group

**Fig. 11:**
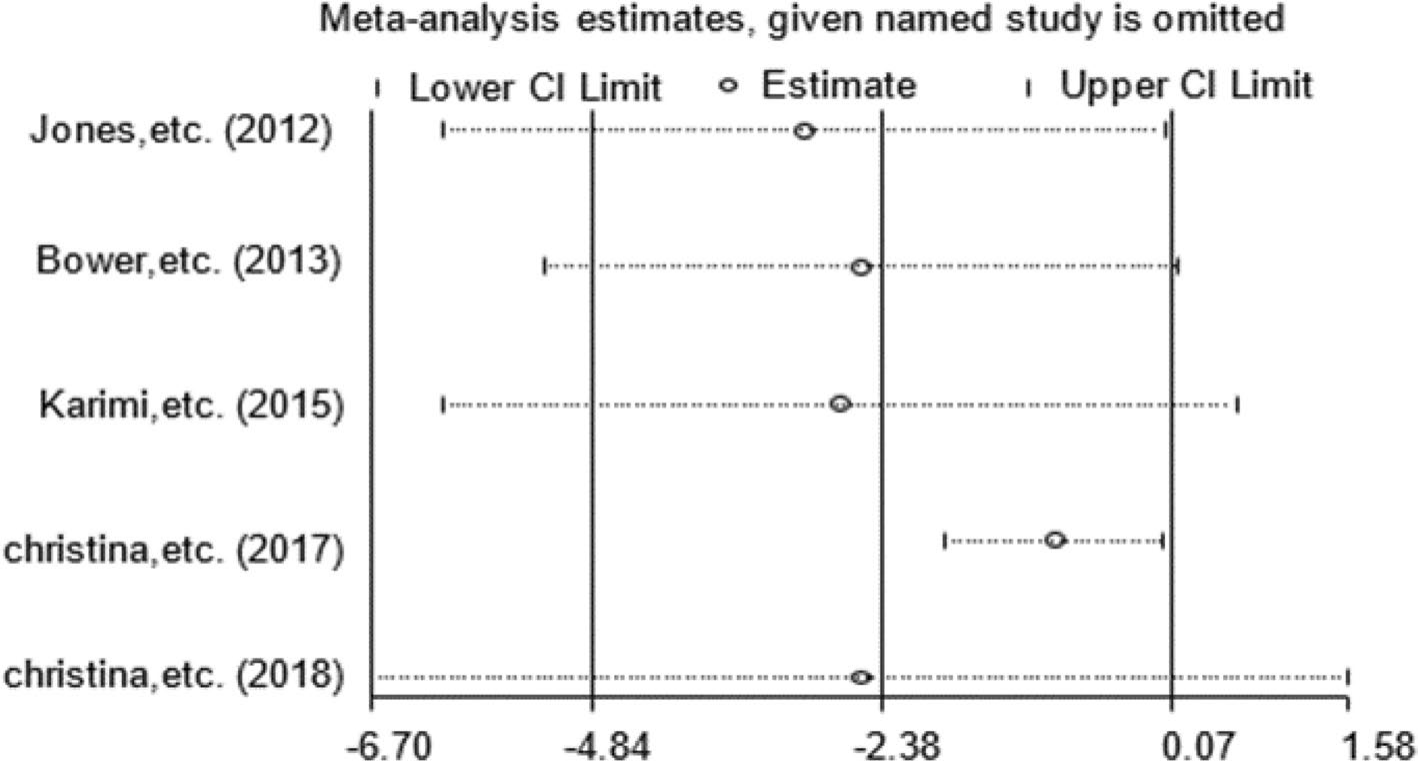
Sensitivity analysis of CRP levels

**Fig. 12:**
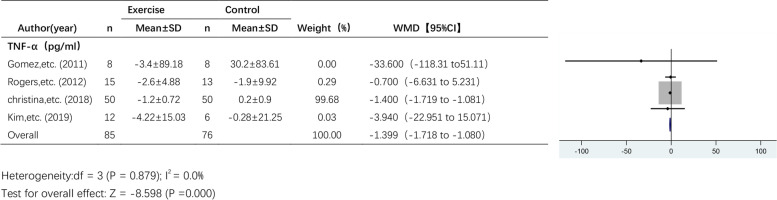
Forest plot of comparison for TNF-α levels after excluding highly heterogeneous studies

Due to the small number of articles, we only performed sensitivity analyses on articles that examined IL-6, TNF-α and CRP levels. The results of the sensitivity analyses of the effects of exercise on IL-6 and CRP levels are shown in Figs. 10 and 11, respectively. The results were found to be relatively stable and reliable. However, sensitivity analysis of the 5 studies that examined TNF-α levels (Table 4) revealed a high degree of heterogeneity in the study by Jones et al. This study was excluded, and meta-analysis was performed again (Fig. 12). There was no heterogeneity (I^2^ = 0.0%), and thus, a fixed effects model was used. The results showed a significant difference in TNF-α levels between the exercise group and the control group (WMD, -1.399 pg/ml; 95% CI, -1.718 to -1.080; *P* = 0.000).

**Table 4:** Sensitivity analysis of CRP

Study omitted	Estimate	[95% CI]
Gomez, et al. (2011)	-.73597163	-1.9933293 to 0 .52138609
Jones, et al. (2012)	-1.3991472	-1.7181057 to -1.0801886
Rogers, et al. (2012)	-.74548692	-2.0388248 to 0.54785085
Christina, et al. (2018)	-.06109226	-.24138363 to 0.11919912
Kim, et al. (2019)	-.72941327	-1.9951227 to 0.53629613
Combined	-.74299052	-1.9951979 to 0.50921682

Jones, etc. (2012) has obvious heterogeneity

### Publication bias

The Begg (*p* = 0.602) test and Egger (*p* = 0.068) test showed no risk of publication bias, and we did not perform a funnel analysis because there were fewer than 10 references.

## Discussion

Most existing studies on the effects of exercise on inflammatory factors and IGF systems in breast cancer survivors examine IL-6, IL-10, IL-1β, TNF-α, CRP, IGF-1, and IGFBP-3 levels. Previous meta-analyses have quantified the effects of exercise on the levels of inflammatory factors and IGF systems [49, 50], but no quantitative analysis was performed based on exercise period and type. The current study explores the effects of different intervention periods and different intervention types on the combined effects of outcomes. Further quantitative evaluation of the effects of exercise on inflammatory factors and IGF systems will provide a basis for more individualized studies.

Our meta-analysis showed that exercise intervention significantly reduced IGF-1 levels in breast cancer survivors, and further subgroup analyses showed significant improvements in IL-6 levels in the exercise group compared with the control group when the intervention period was longer than 12 weeks and a significant effect of exercise on CRP and IL-10 levels when the intervention period was less than or equal to 12 weeks. In addition, aerobic exercise plus resistance training significantly reduced IL-6 levels. Sensitivity analysis showed that exercise had a significant effect on TNF-α levels in breast cancer survivors after excluding highly heterogeneous studies.

### Inflammatory factors

Tumour-associated inflammation is one of the hallmarks of breast cancer [51]. Studies have shown that exercise is associated with lower levels of various proinflammatory cytokines [22, 23]. A study on breast cancer patients found a negative association between exercise and IL-6 levels; other meta-analyses have also shown a significant decrease in IL-6 and CRP levels after exercise intervention [52]. However, in our meta-analysis, we only found that exercise intervention significantly reduced TNF-α levels after excluding highly heterogeneous studies; we did not find a significant effect of exercise on any other inflammatory factors. Further subgroup analyses showed that exercise significantly reduced IL-6 levels when the intervention period was > 12 weeks, but exercise had a nonsignificant effect on IL-6 levels when the intervention period was ≤ 12 weeks. In contrast, exercise significantly reduced CRP and IL-10 levels when the intervention period was ≤ 12 weeks. This suggests a possible effect of different intervention periods on the experimental results. There was significant heterogeneity in the results of the intervention period subgroup analysis (91.3%, 99.5%, 98.0%, 60.7%), suggesting that the effect of exercise on IL-6, CRP, TNF-α, and IL-10 levels in breast cancer patients with different intervention periods is likely to be a source of heterogeneity in the included studies. Therefore, we hypothesized that there may be a complex correlation between the period of intervention and changes in inflammatory factors. A study analysis [52] concluded that for the elderly population, short-term intervention training hardly leads to significant changes in the organism at the level of indicators. However, long-term exercise may not show changes in inflammatory markers, as older adults are susceptible to other uncontrolled environmental factors, which in turn affect the levels of inflammatory factors. In addition, we found that aerobic plus resistance training significantly reduced IL-6 levels. Previous studies [53, 54] have identified the benefits of aerobic plus resistance training, which improves the overall functional capacity of breast cancer patients. As a result, a growing number of studies have now evolved from just one type of exercise to a more complex exercise prescription of aerobic exercise combined with resistance training. However, we included too few studies on other inflammatory factors to analyse the effects of other intervention types on outcomes. More studies should be included in the future to analyse the effects of the exercise intervention period and exercise type on inflammatory markers in breast cancer patients to develop the optimal intervention.

### IGF system

The IGF system is an important mechanism in breast cancer pathogenesis. The IGF signalling system plays an important role in breast cancer development and progression [55, 56]. Therefore, targeting the IGF system is a better option. Studies have shown that exercise can reduce IGF-1 levels and increase IGFBP levels [28]. Exercise is thought to cause physiological changes in systemic IGF ligand and binding protein bioavailability, which may indirectly affect IGF-1R signalling [57]. In our meta-analysis, we included three studies that examined IGF-1 levels, and all three studies reported that exercise significantly decreased IGF-1 levels. A meta-analysis [50] involving 7 studies of breast cancer patients also found a trend towards decreased IGF-I levels after exercise, but this was not statistically significant (WMD, -5.23 ng/mL; 95% CI, 13.00 to 2.53; *p* = 0.19). However, this meta-analysis found no significant effect of exercise on IGFBP-3 levels (WMD, 0.01; 95% CI, -0.96 to 0.98; *p* = 0.99). This is similar to the findings of our study, which found a nonsignificant trend of improved IGFBP-3 levels after exercise. Among the included studies that examined levels IGFBP-3 that we included, two of them [42, 46] reported elevated IGFBP-3 levels after exercise, while another 6-month aerobic exercise study [49] found a significant reduction of 4.1% (*p* = 0.006) in IGFBP-3 levels in the intervention group compared to the control group. The results showed a significant discrete pattern, which may be related to intervention type. In a 6-month study [58] of prostate cancer patients with aerobic or resistance exercise programs, a significant increase in serum IGFBP-3 levels (12.1%; P ≤ 0.05) was observed in the resistance exercise group, while IGFBP-3 levels were reduced by 23.7% (P ≤ 0.05) in the aerobic exercise group. However, this may be only a conjecture, as baseline levels of IGFBP-3 were significantly higher in the aerobic exercise group than in the resistance training group in this study, and participants in this study also differed significantly from those in our study. Therefore, more studies should be included in the future meta-analyses.

The meta-analysis had a small number of included studies and included only English and Chinese studies, which may have led to selection bias. Second, this study did not adjust for potential confounders such as age, BMI, and sex, which may have a potential effect on the study results. Third, due to the small number of included studies and the lack of available data, the subgroup analysis only examined the effect of different exercise periods and types on certain outcomes. A comprehensive subgroup analysis of the characteristics of exercise intervention is warranted for future studies.

## Conclusion

This study affirms that exercise positively affects inflammatory factors and the IGF system in breast cancer survivors. Exercise is feasible for breast cancer survivors. However, the most beneficial exercise period, type and intensity for improving inflammatory factors and IGF systems in breast cancer patients are not clear. Future studies should include more randomized controlled trials to analyse the appropriate exercise intervention for breast cancer patients to improve inflammatory factors and IGF systems and to provide a basis for developing individualized exercise prescriptions for breast cancer patients.

## Supplementary Information

**Additional file 1.**

## Data Availability

All data generated or analyzed during this study are included in this published article [and its supplementary information files].

## Abbreviations

AT: Aerobic training
RT: Resistance training
HE: Health education
GS: Ginger supplement
SST: Standard support therapy
UC: Usual care
TCC: Tai chi chuan
