# Supplementary material for: Effects of exercise on inflammatory factors and IGF system in breast cancer survivors: a meta-analysis
Source: BMC Womens Health. 2022 Dec 8;22:507. doi: 10.1186/s12905-022-02058-5 (PMC9730577; doi:10.1186/s12905-022-02058-5)
Supplement: Supplementary file 1 — Additional file 1. [file 12905_2022_2058_MOESM1_ESM.docx]

The search terms we have applied are combinations of the terms we have listed. **Total 108 search terms. As follows:**

physical activity and breast cancer and inflammatory，physical activity and breast cancer and IL-6, physical activity and breast cancer and IL-10, physical activity and breast cancer and IL-1β, physical activity and breast cancer and CRP, physical activity and breast cancer and TNF-α，physical activity and breast cancer and IGF，physical activity and breast cancer and IGF-1，physical activity and breast cancer and IGFBP-3,

physical activity and breast tumor and inflammatory，physical activity and breast tumor and IL-6, physical activity and breast tumor and IL-10, physical activity and breast tumor and IL-1β, physical activity and breast tumor and CRP, physical activity and breast tumor and TNF-α，physical activity and breast tumor and IGF，physical activity and breast tumor and IGF-1，physical activity and breast tumor and IGFBP-3,

physical activity and breast oncology and inflammatory，physical activity and breast oncology and IL-6, physical activity and breast oncology and IL-10, physical activity and breast oncology and IL-1β, physical activity and breast oncology and CRP, physical activity and breast oncology and TNF-α，physical activity and breast oncology and IGF，physical activity and breast oncology and IGF-1，physical activity and breast oncology and IGFBP-3，

exercise and breast cancer and inflammatory，exercise and breast cancer and IL-6, exercise and breast cancer and IL-10, exercise and breast cancer and IL-1β, exercise and breast cancer and CRP, exercise and breast cancer and TNF-α，exercise and breast cancer and IGF，exercise and breast cancer and IGF-1，exercise and breast cancer and IGFBP-3,

exercise and breast tumor and inflammatory，exercise and breast tumor and IL-6, exercise and breast tumor and IL-10, exercise and breast tumor and IL-1β, exercise and breast tumor and CRP, exercise and breast tumor and TNF-α，exercise and breast tumor and IGF，exercise and breast tumor and IGF-1，exercise and breast tumor and IGFBP-3,

exercise and breast oncology and inflammatory，exercise and breast oncology and IL-6, exercise and breast oncology and IL-10, exercise and breast oncology and IL-1β, exercise and breast oncology and CRP, exercise and breast oncology and TNF-α，exercise and breast oncology and IGF，exercise and breast oncology and IGF-1，exercise and breast oncology and IGFBP-3,

sport and breast cancer and inflammatory，sport and breast cancer and IL-6, sport and breast cancer and IL-10, sport and breast cancer and IL-1β, sport and breast cancer and CRP, sport and breast cancer and TNF-α，sport and breast cancer and IGF，sport and breast cancer and IGF-1，sport and breast cancer and IGFBP-3,

sport and breast tumor and inflammatory，sport and breast tumor and IL-6, sport and breast tumor and IL-10, sport and breast tumor and IL-1β, sport and breast tumor and CRP, sport and breast tumor and TNF-α，sport and breast tumor and IGF，sport and breast tumor and IGF-1，sport and breast tumor and IGFBP-3,

sport and breast oncology and inflammatory，sport and breast oncology and IL-6, sport and breast oncology and IL-10, sport and breast oncology and IL-1β, sport and breast oncology and CRP, sport and breast oncology and TNF-α，sport and breast oncology and IGF，sport and breast oncology and IGF-1，sport and breast oncology and IGFBP-3,

training and breast cancer and inflammatory，training and breast cancer and IL-6, training and breast cancer and IL-10, training and breast cancer and IL-1β, training and breast cancer and CRP, training and breast cancer and TNF-α，training and breast cancer and IGF，training and breast cancer and IGF-1，training and breast cancer and IGFBP-3,

training and breast tumor and inflammatory，training and breast tumor and IL-6, training and breast tumor and IL-10, training and breast tumor and IL-1β, training and breast tumor and CRP, training and breast tumor and TNF-α，training and breast tumor and IGF，training and breast tumor and IGF-1，training and breast tumor and IGFBP-3,

training and breast oncology and inflammatory，training and breast oncology and IL-6, training and breast oncology and IL-10, training and breast oncology and IL-1β, training and breast oncology and CRP, training and breast oncology and TNF-α，training and breast oncology and IGF，training and breast oncology and IGF-1，training and breast oncology and IGFBP-3.
